# Use of Circulating Tumour DNA (ctDNA) for Measurement of Therapy Predictive Biomarkers in Patients with Cancer

**DOI:** 10.3390/jpm12010099

**Published:** 2022-01-13

**Authors:** Michael J. Duffy, John Crown

**Affiliations:** 1 UCD School of Medicine, Conway Institute of Biomolecular and Biomedical Research, University College Dublin, D04 V1W Dublin, Ireland; 2 UCD Clinical Research Centre, St. Vincent’s University Hospital, D04 T6F4 Dublin, Ireland; 3 Department of Medical Oncology, St Vincent’s University Hospital, D04 T6F4 Dublin, Ireland; John.Crown@ccrt.ie

**Keywords:** biomarker, predictive, companion diagnostic, ctDNA, liquid biopsy, cancer

## Abstract

Biomarkers that predict likely response or resistance to specific therapies are critical in personalising treatment for cancer patients. Such biomarkers are now available for an increasing number of anti-cancer therapies, especially targeted therapy and immunotherapy. The gold-standard method for determining predictive biomarkers requires tumour tissue. Obtaining tissue, however, is not always possible and even if possible, the amount or quality of tissue obtained may be inadequate for biomarker analysis. Tumour DNA, however, can be released into the bloodstream, giving rise to what is referred to as circulating tumour DNA (ctDNA). In contrast to tissue, blood can be obtained from effectively all patients in a minimally invasive and safe manner. Other advantages of blood over tissue for biomarker testing include a shorter turn-around time and an ability to perform serial measurements. Furthermore, blood should provide a more complete profile of mutations present in heterogeneous tumours than a single-needle tissue biopsy. A limitation of blood vis-à-vis tissue, however, is lower sensitivity and, thus, the possibility of missing an actionable mutation. Despite this limitation, blood-based predictive biomarkers, such as mutant *EGFR* for predicting response to EGFR tyrosine kinase inhibitors in advanced non-small-cell lung cancer and mutant *PIK3CA* for predicting response to alpelisib in combination with fulvestrant in advanced breast cancer, may be used when tissue is unavailable. Although tissue remains the gold standard for detecting predictive biomarkers, it is likely that several further blood-based assays will soon be validated and used when tissue is unavailable or unsuitable for analysis.

## 1. Introduction

Circulating tumour DNA (ctDNA), i.e., DNA shed by malignant cells into the circulation, is emerging as potentially one of the most transformative biomarkers in oncology [1,2,3]. Indeed, preliminary results suggest that measurement of ctDNA may be useful in screening for early cancer, detecting micrometastasis (minimum residual disease) following curative-intent surgery, upfront selecting appropriate systemic treatment and monitoring response to ongoing therapy in advanced disease [1,2,3]. Although its role in most of these setting is still largely exploratory, ctDNA measurement has already entered clinical use for systemic therapy selection.

Rationally, selecting treatment is aided by biomarkers known as predictive or response biomarkers. Such tests upfront identify patients that are likely to be responsive or resistant to a specific treatment. A related type of biomarker, known as a companion diagnostic, is defined by US Food and Drug Administration (FDA) as a medical device that provides information that is essential for the safe and effective use of a corresponding drug or biological product.

Predictive biomarkers/companion diagnostics are important in therapy decision making as they help to identify patients that are likely to benefit from a specific therapy. Consequently, such patients can then be treated with a relevant therapy with a high probability of success. At the same time, these biomarkers can spare patients who are unlikely to be responsive, from unnecessary drug toxicity and costs. In some situations, these patients may be able to receive a different effective therapy, thus saving time and money. Alternatively, such patients may be able to participate in an appropriate clinical trial. Overall, the availability of predictive biomarkers/companion diagnostics is leading to a more rational, cost-effective and personalised use of anti-cancer therapies. Importantly, in an increasing number of situations, performance of these tests when followed by administration of the appropriate matching therapy is resulting in better response rates, improved outcome and less therapy-induced toxicity than was traditionally available [4,5,6,7,8,9,10,11,12].

## 2. Current Strategies for Measurements of Predictive Biomarkers/Companion Diagnostics

Predictive biomarkers/companion diagnostics are currently available for a broad range of systemic therapies, especially targeted therapies and immunotherapy with immune check point inhibitors (Table 1) [13,14,15,16]. Indeed, the vast majority of new therapies currently entering clinical use has an associated predictive biomarker/companion diagnostic. The gold standard methodology for measuring predictive biomarkers/companion diagnostics requires tumour tissue. Acquiring tissue, however, necessitates an invasive procedure, such as surgery or biopsy, which is inconvenient for patients and in some situations may cause harm. Indeed, in some elderly or frail patients, a biopsy may not be possible and even if possible, it may not yield a sufficient amounts of suitable tissue for biomarker analysis [17,18,19]. Obtaining tissue may be especially difficult in patients with advanced progressing therapy-resistant cancers or in patients with certain sites of metastases (e.g., brain). Thus, in one report, a biopsy could not be performed in 50% of the patients with non-small-cell lung cancer (NSCLC) who developed resistance to first-line EGFR tyrosine kinase inhibitors (TKI) [18]. Even if tissue is accessed, it may not be sufficient or suitable for measuring the multiple biomarkers currently recommended for specific cancers such as NSCLC. Indeed, in some reports less than 20% of biopsies were found to have adequate tissue for detecting all the recommended genomic tests in patients with NSCLC [20].

A further potential problem with tissue is molecular intra- and inter-tumour heterogeneity. DNA sequencing using multi-locational sampling has shown that malignant tissue is composed of highly heterogeneous cell populations as regards to somatic mutations [21]. Single biopsies whether from a primary or metastatic tumour may thus not be representative of the whole tumour load but rather indicative of genomic state at a single time point and at a single location. Heterogeneity is likely to be most prevalent in patients with rapidly evolving tumours, multi-focal tumours or multiple metastases and in those previously treated who develop acquired resistance to the therapy. Indeed, in the setting of acquired therapy resistance, the emergence of multiple resistance mechanisms appears to be widespread [22]. Clearly, in such a situation, a single biopsy may miss potentially actionable mutations. Other limitations of tissue for analysis of predictive biomarker companion diagnostics are listed in Table 2.

## 3. Advantages of Blood over Tissue for Measuring Predictive Biomarkers/Companion Diagnostics

Many of the limitations of tissue for biomarker testing can be circumvented by measuring DNA shed from tumours into blood (Table 2). Use of circulating ctDNA (also known as liquid biopsy) is thus an attractive alternative source of tumour DNA, when surgery/biopsy cannot be performed or when the tissue obtained is insufficient or unsuitable for analysis [1,2,3]. Furthermore, use of ctDNA might be expected to give a broader overview of mutations present at different tumour locations than a single-needle biopsy, thus potentially detecting more actionable mutations [22,23]. Indeed, across different tumour types, use of ctDNA has resulted in the detection of more potentially actionable genes than tissue [23,24,25,26]. Other key advantages of ctDNA over tissue testing include faster turn-around time for reporting of result [26,27], less costly to perform [28,29] and ease of performing serial measurements. The latter may be particularly useful in monitoring the effectiveness of ongoing treatments, identifying the early emergence of resistance and identifying the mechanism of resistance, see below.

These advantages of blood over tissue have led in recent years to the intensive investigation of ctDNA for the measurement of predictive biomarker/companion diagnostic, for a range of therapeutics across different cancer types. Indeed, several therapeutics currently have approved ctDNA-based assays for guiding their use (see below). The clinical utility of these approved ctDNA assays is discussed below. In addition, we also discuss emerging plasma-based predictive biomarkers/companion diagnostics.

## 4. Mutant *EGFR* for Predicting Response to EGFR TKIs in Patients with Non-Small-Cell Lung Cancer

The first ctDNA predictive biomarker/companion diagnostic to enter clinical use involved mutational analysis of *EGFR* for the identification of patients with NSCLC that were likely to benefit from EGFR tyrosine kinase inhibitors (TKIs) [30,31,32]. Activating (also known as sensitising) mutations somatic *EGFR* mutations are present in 15–25% of Caucasian subjects and in 35–50% of East-Asian patients with untreated NSCLC [30,31,32]. Approximately 90% of these mutations involve an in-frame deletion in exon 19 (codons 746–750) or an L858R point mutation in exon 21. Less frequent mutations include G719X (4%), L861X (1%), S768I (1%) mutations, and exon 20 insertions (2%). These mutations, especially the in-frame deletion in exon 19 and L858R, alter the structure of the ATP binding pocket in the tyrosine kinase domain of EGFR, leading to ligand-independent activation. This in turn results in increased RAS-RAF-MAPK signalling, which ultimately drives the growth and progression of NSCLC.

Since mutant *EGFR* is a driver gene for a subset of NSCLC, several TKI were developed to target the corresponding mutant protein. Currently, at least five clinically approved (US FDA) EGFR TKI are available, gefitinib, erlotinib (first generation), afatinib and dacominitib (second generation) and osimertinib (third generation) [32]. Gefitinib, erlotinib and afatinib are selective for *EGFR* sensitising mutation such as the in-frame deletion in exon 19 or L858R point mutations in exon 21, while osimertinib is selective for both the sensitising mutations and T790M resistance mutation (see below). Since these compounds possess higher affinity than the natural kinase substrate, ATP for the ATP-binding pocket in EGFR, they competitively inhibit the tyrosine kinase activity of the mutant receptor, thus blocking downstream signalling which in turn leads to inhibition of tumour cell growth.

Several randomised phase III trials have now shown that treatment of NSCLC patients with advanced disease possessing sensitising *EGFR* mutations using first- or second-generation TKIs resulted in superior response rates, enhanced progression-free survival, reduced toxicities and increased quality of life compared to standard chemotherapy (for review, see references [30,31,32,33]). Recently, the third generation TKI inhibitor, osimertinib was shown to enhance overall survival compared with gefitinib or erlotinib (hazard ratio (HR) for death, 0.80; 95.0% CI, 0.64 to 1.00; *p* = 0.046) [34]. Based on this finding, joint guidelines recently published by the American Society of Clinical Oncology (ASCO) and Ontario Health (OH) stated that where available, osimertinib should be the optimal first-line treatment for NSCLC patients with sensitising *EGFR* mutations [35].

Based on the early clinical results with first generation TKIs, tissue mutation analysis of *EGFR* entered clinical use for selecting patients with metastatic NSCLC likely to benefit from these drugs. However, as mentioned above, the use of tissue has several limitations, many of which can be largely overcome by analysing ctDNA. Overall, strong concordance (70–90%) is found between the presence of *EGFR* activating mutations in tumour tissue and matching ctDNA [36,37,38,39,40]. Thus, following a meta-analysis of 27 studies involving > 4000 patients, the pooled sensitivity, specificity and areas under the curve for ctDNA versus tissue were 60%, 94%, and 0.9208 for the detection of *EGFR* mutations. For detecting exon 19 deletions, the corresponding values were 64%, 99%, and 0.9583 and for detecting L858R mutations, values were 57%, 99%, and 0.9605, respectively [36]. In a more recent prospective study involving 180 patients with advanced NSCLC, ctDNA had a specificity of 100% for both *EGFR* 19 del and L858R mutation [37]. The sensitivity of ctDNA however, was 82% for *EGFR* 19 del and 74% for L858R. For a commercial *EGFR* ctDNA mutation assay approved by both the European Medicines Agency (EMA) and the US Food and Drug Administration (FDA), i.e., Cobas (Roche Molecular Systems) (see below), the ctDNA assay exhibited a specificity of 98.2% and a sensitivity of 76.7% for the detection of both the *EGFR* exon 19del and L858R mutations [38]. These latter results were achieved with patients participating in a randomised phase III trial (ENSURE) which compared the efficacy and safety of erlotinib with gemcitabine + cis-platin in patients with advanced NSCLC [39].

Clearly, ctDNA provides high specificity for detecting *EGFR* activating mutations across multiple different studies. Sensitivity of ctDNA assays however, is less good. Despite this lower sensitivity using ctDNA, both assays have been reported to have similar predictive value for evaluating response to EGFR TKIs [40,41,42].

The high specificity of ctDNA for detecting tumour *EGFR* mutations provides confidence that a positive ctDNA test could be used in selecting NSCLC patients for treatment with EGFR TKIs. However, because of the relatively low sensitivity (70–80%), absence of a plasma mutation does not necessary exclude the presence of a mutation in the corresponding tumour specimen. In such a situation, tissue should be evaluated when feasible. Based on its high specificity [37,38,39,40], minimally invasive approach as well as a faster turn-around-time for reporting [27,28], several expert panels currently recommend that for patients with advanced NSCLC where tissue cannot be obtained or is insufficient for mutation testing, ctDNA may be used to identify *EGFR* mutations [43,44,45,46]. The guidelines, however, also add that because the sensitivity of ctDNA assays is lower than that of tissue, a negative result from a ctDNA test should not be interpreted that the corresponding tumour is also *EGFR* mutation-negative [43,44,45,46]. As mentioned above, in such a situation, tissue testing should be performed where possible.

Several different research and commercial assays are available for detecting *EGFR* mutation in ctDNA, including real-time/quantitative PCR, digital droplet (dd) PCR, BEAMing PCR and next generation sequencing (NGS) (for detailed review, see reference [46]). Importantly, from a clinical point of view, some of the commercially available assays have received regulatory approval in different parts of the world. One of the first to receive such approval was the cobas EGFR Mutation Test v2. This real-time PCR assay which is approved by both the EMA and the US FDA, detects 42 mutations in exons 18–21 of the *EGFR* gene, including L858R, exon 19 deletions, L861Q and T790M. Another PCR test, i.e., the therascreen EGFR Plasma RGQ PCR Kit (Qiagen), has received the Conformité Européenne (CE) mark for use in Europe. This assay detects deletions in exon 19, the T790M mutation in exon 20 and the L858R mutation in exon 21 of the *EGFR* gene.

While PCR is suitable for detecting single/low number of mutations, it is not appropriate for analysing the full panel of biomarkers currently recommended for patients with NSCLC. Thus, the 2021 guidelines recently published by the International Association for the Study of Lung Cancer (IASLC) state “because of the growing number of guideline-recommended oncogene targets to be assessed in advanced NSCLC, testing of plasma ctDNA should be performed by a clinically validated NGS platform rather than single-gene, PCR-based approaches, both in treatment-naive patients and those associated with multiple mechanisms of acquired resistance to targeted agents”. However, the group also added that if NGS is not available, single-gene analysis by PCR may be used. Next generation sequencing tests approved by the US FDA for predicting response to EGFR TKIs in NSCLC include the Guardant360 CDx and the FoundationOne Liquid CDx, assays [46].

## 5. T790M Mutant *EGFR* for Predicting Acquired Resistance to First Generation EGFR TKIs in Patients with Non-Small-Cell Lung Cancer

While *EGFR* sensitising mutations such as L858R and exon 19 deletions are associated with benefit from EGFR TKIs in patients with NSCLC, acquired resistance at least to gefitinib and erlotinib occurs with the emergence of the T790M mutation. This secondary mutation which is found in 50 to 60% of resistant cases [47] is believed to cause resistance by increasing the affinity of EGFR for its physiological substrate, ATP, thus sterically blocking the binding of first generation TKIs [47]. Most patients with this mutation, however, respond to the third generation EGFR TKI, osimertinib [47].

As with the more commonly occurring *EGFR* activating mutations, the *EGFR* T790M resistance mutation can also be detected using ctDNA. Overall, the concordance between tissue and ctDNA for the T790M mutation is less good that is found with the exon 19 deletions and L858R mutations [47,48,49,50]. Thus, following a systematic review of 21 studies involving 1639 patients, the pooled sensitivity of ctDNA versus tissue was 67%, the pooled specificity was 80% while the pooled positive predictive value (PPV) was 85% and the pooled negative predictive value (NPV) was 60% [50]. The lower predictive accuracy of ctDNA is possibly due to the T790M mutation being an acquired mutation and as a result is subclonal and present at lower abundance than the activating driver mutations.

Despite this less than ideal concordance, response rates to osimertinib in patients positive for the ctDNA T790M mutations appear to be similar to those positive using a tissue biopsy [48,49,50,51]. Thus, as with the L858R mutation and exon 19 deletions, several expert panels currently state that ctDNA may be used to identify *EGFR* T790M mutations for predicting response to osimertinib in patients with advanced NSCLC who develop acquired resistance to first generation EGFR-TKIs [43,44,45,46]. Again, the guidelines add that testing of tumour tissue if feasible is recommended, if the ctDNA result is negative. Going further, the IASLC state that when acquired resistance to TKI occurs, “initial use of ctDNA is preferred for evaluating mechanisms of resistance, with repeat biopsy if ctDNA is uninformative” [46].

## 6. *EGFR* Exon 20 Insertions for Predicting Response to Amivantamab-Vmjw or Mobocertinib

Two to 3% of NSCLC patients possess an *EGFR* exon 20 insertion which confers resistance to the currently approved EGFR TKIs [51]. Patients with this mutation, however, were recently reported to respond to amivantamab-vmjw (a bispecific antibody that targets both EGFR and MET) [52] or mobocertinib (a tyrosine kinase inhibitor) [53]. Subsequently, both these therapies were approved by the US FDA for the treatment of NSCLC patients with an *EGFR* exon 20 insertion. Simultaneously with the approval of amivantamab-vmjw, the US FDA also approved a companion diagnostic (Guardant360 CDx) that may be measured using ctDNA to identify patients with *EGFR* exon 20 insertions.

## 7. Mutant *PIK3CA* for Predicting Response to Alpelisib in Patients with Advanced Breast Cancer

Another approved ctDNA test for predicting response to a specific therapy involves mutation analysis of *PIK3CA* for identifying patients with advanced breast cancer likely to benefit from treatment with the α-specific PIK3CA inhibitor, alpelisib (in combination with the oestrogen receptor degrading compound, fulvestrant) [54].

The *PIK3CA* gene, which codes for the catalytic subunit of the PI3K signalling protein, is the most frequently mutated oncogene in breast cancer, with mutations present in 35–40% of samples [55,56]. Most of the *PIK3CA* mutations occur at one of three hotspots, i.e., at E545K and E542K in exon 9 within the helical domain and at H1047 in exon 20 within the kinase domain. Although the vast majority of mutations in *PIK3CA* are single mutations, 12–15% of breast cancers have multiple mutations in the gene [57]. The presence of multiple cis mutations has been reported to increase oncogenicity [57].

Analogous to the situation with mutant *EGFR* in patients with NSCLC, a strong correlation has been found between the presence of *PIK3CA* mutations in breast tumour tissue and corresponding ctDNA. For example, a meta-analysis of 7 cohorts from 5 studies involving 247 patients, concluded that the pooled sensitivity and specificity of ctDNA for detecting tissue *PIK3CA* mutations were 86% and 98%, respectively [58]. In 2019, the US FDA approved the administration of alpelisib in combination with fulvestrant for the treatment of metastatic breast cancer patients with hormone receptor-positive, HER2-negative breast cancer possessing *PIK3CA*-mutations, following disease progression on or after endocrine therapy [54]. This approval was based on findings from a randomised phase III trial (SOLAR-I trial) which compared treatment with alpelisib plus fulvestrant versus treatment with fulvestrant alone in patients with hormone receptor-positive, HER2-negative patients that had previously progressed on endocrine therapy [59]. The trial showed that alpelisib in combination with fulvestrant almost doubled the median progression-free survival compared with fulvestrant alone (median progression free survival, 11.0 months versus 5.7 months; HR = 0.65, 95% CI: 0.50–0.85; *p* < 0.001) in patients with *PIK3CA* mutations (detected in tissue or plasma) but had no benefit in those lacking such mutations. In addition to progression-free survival, overall response rates were also significantly increased when alpelisib was combined with fulvestrant in patients with *PIK3CA* mutations (35.7% for the combination versus 16.2% for fulvestrant alone; *p* = 0.0002). This extended overall survival was confirmed in a recent update report which showed an 8-month improvement in median overall survival with alpelisib plus fulvestrant versus fulverstrant monotherapy in patients with *PIK3CA*-mutated tumours (although the findings did not cross the pre-specified boundary for statistical significance) [60].

Simultaneously with the approval of alpelisib + fulvestrant for advanced breast cancer treatment, the US FDA also approved a companion diagnostic test, i.e., therascreen PIK3CA RGQ PCR Kit, for detecting *PIK3CA* mutation in tissue and/or ctDNA. This assay detects 11 *PIK3CA* hotspot mutations, i.e., exon 7: C420R; exon 9: E542K, E545A, E545D [1635G > T only], E545G, E545K, Q546E, Q546R; and exon 20: H1047L, H1047R, H1047Y. (Available online: https://www.accessdata.fda.gov/cdrh_docs/pdf19/P190001A.pdf, accessed on 1 November 2021). According to the FDA approval statement, breast cancer patients with tissue or plasma-positive therascreen PIK3CA RGQ PCR Kit test result are eligible for treatment with alpelisib. However, in patients with plasma-negative results, reflexing to tumour tissue testing for the presence of *PIK3CA* mutations should be performed if possible. More recently, the Foundation Medicine’s FoundationOne CDx (F1CDx) assay also received US FDA approval for predicting benefit from alpelisib in breast cancer. This test detects the same 11 mutations as the QIAGEN assay mentioned above.

In addition to alpelisib, the ability of circulating mutant *PIK3CA* to predict response to other PIK3CA inhibitors has also been investigated. In the SANDPIPER phase III clinical trial which compared the efficacy of the α, γ and δ PIK3CA inhibitor, taselisib in combination with fulvestrant, versus a placebo + fulvestrant, the presence of *PIK3CA* mutations also predicted an enhanced response to the PI3KCA inhibitor-based treatment, i.e., HR for progression free survival for patient with *PIK3CA*-mutant-positive was 0.86 (95% CI, 0.57–1.27) compared to 0.62 (95% CI, 0.47–0.83) in *PIK3CA* mutation-negative patients [61]. However, patients with two or more mutations in *PIK3CA* had a superior progression free survival compared to those with only one mutation, i.e., HR of 0.37 versus 0.68. It should be stated however, that although this trial met its primary endpoint, taselisib plus fulvestrant was deemed to lack clinical utility due its toxicity and modest clinical benefit [61].

## 8. Mutant *RAS* for Predicting Resistance to Anti-EGFR Antibodies in Patients with Colorectal Cancer

Mutation testing of *RAS* (*KRAS* and *NRAS*) in tumour tissue has been routinely used for several years to identify patients with advanced CRC who are intrinsically resistant to anti-EGFR antibodies (cetuximab, panitumumab) [62]. Thus, the most recent guidelines published jointly by the American Society for Clinical Pathology, College of American Pathologists, Association for Molecular Pathology, and the American Society of Clinical Oncology state that colorectal carcinoma patients being considered for anti-EGFR therapy must undergo *RAS* mutational testing. Mutational analysis should include *KRAS* and *NRAS* codons 12, 13 of exon 2; 59, 61 of exon 3; and 117 and 146 of exon 4 [63].

As with *EGFR* mutations in advanced NSCLC and *PIK3CA* mutations in advanced breast cancer, strong concordance has been found between the *RAS* mutational status of CRC tissue and matching plasma in patients with advanced CRC [62,64,65,66]. Thus, across seven independent studies, concordance between the two sites was found to vary from 78% to 96%, while the sensitivities and specificities of ctDNA varied from 72% to 96% and 83% and 98%, respectively [62]. Lower sensitivity was found in patients with nodal, peritoneal or lung metastases as well as in those with mucinous-type tumours [62].

Consistent with this high concordance, retrospective studies have reported that the outcome of patients with advanced CRC treated with cetuximab or panitumumab was similar, irrespective of whether *RAS* mutation testing was performed using tissue or plasma [64,65,66]. We should however, mention that although most reports showed similar predictive value for ctDNA and tissue assays, a small exploratory study concluded that a positive ctDNA *RAS* mutations result did not preclude response to panitumumab, i.e., 11% of patients with a plasma-positive test had either a complete or partial response to the antibody [67]. Clearly, the findings in this provocative preliminary report require confirmation.

Despite the overall good concordance between tissue and plasma, most expert panels continue to recommend that *RAS* mutation assays for predicting response to anti-EGFR antibodies in CRC are performed on tissue. However, according to the European Society of Digestive Oncology (ESDO) expert panel, a ctDNA assay could be used when obtaining tissue is technically or logistically not possible [68]. As well as ESDO, the Japanese Society of Medical Oncology recommend ctDNA testing for “evaluating the suitability” of anti-EGFR therapy in patients with unresectable CRC [69]. One of the obstacles to having a clinically useful ctDNA assay for ctDNA *RAS* mutations is lack of prospective validation and lack of a validated cut-off point for *RAS* mutations.

One of the first commercially available ctDNA for detecting mutations in *RAS* to have received regulatory approval was OncoBEAM™ RAS CRC which has obtained the EU CE Mark IVD status (Sysmex). This test uses a form of digital PCR (BEAMing technology) to detect 34 mutations in *KRAS* and *NRAS* [70].

Although CRC patients with wild-type RAS may initially benefit from anti-EGFR antibody therapy, acquired resistance is inevitable and usually occurs within the first year of treatment. One of the most frequent mechanisms of acquired resistance involves secondary mutations in *RAS* (*KRAS* and *NRAS*) which are found in approximately one third of cases [71]. However, alteration in other genes, including mutations in *EGFR* (extracellular domain), *BRAF* (V600E), *MEK* as well as amplification of *HER2* and *MET*, has also been associated with the development of acquired resistance [72,73]. Although all of these alterations have been detected in ctDNA [72,73], they are not currently actionable.

Interestingly, cessation of anti-EGFR therapy, followed by a break from these drugs, leads to a decrease in ctDNA *RAS* mutations, opening up the possibility for rechallenge with anti-EGFR therapy. To test this hypothesis, Cremolini et al. [74] initiated a single-arm phase II trial to evaluate the efficacy of cetuximab plus irinotecan for third-line treatment of patients with *RAS* and *BRAF* wild-type metastatic CRC who were initially sensitive to and then developed resistance to the therapy. After a median follow-up of 15.4 months, patients with *RAS* wild-type ctDNA (*n* = 13) were found to have a significantly longer progression-free survival than those with ctDNA positive for *RAS* mutations (*n* = 12). The corresponding median progression-free survival periods were 4.0 and 1.9 months, respectively (HR, 0.44; 95% CI, 0.18–0.98; *p* = 0.03). At time of follow-up, no significant difference was found in overall survival between the groups. In a further phase II trial (CAVE trial), rechallenge with cetuximab and avelumab (an anti PD-L1 antibody) was also found to provide more benefit in ctDNA wild-type than ctDNA KRAS mutant patients (median overall survival, 17.3 months versus 10.4 months [75]. Clearly, these results now require confirmation in prospective randomised trials.

## 9. Other ctDNA Predictive Biomarkers and Companion Diagnostics

In recent years, the USA FDA approved several new companion diagnostics for identifying patients with different types of metastatic cancer likely to benefit from specific therapies. These included:

- The FoundationOne Liquid CDx test for mutant *BRCA1* and *BRCA2* in patients with ovarian cancer eligible for treatment with the PARP inhibitor, rucaparib; *ALK* rearrangements in patients with NSCLC eligible for treatment with the ALK inhibitor, alectinib and mutant *BRCA1, BRCA2* and *ATM* in patients with metastatic castration-resistant prostate cancer eligible for treatment with the PARP inhibitor, olaparib.
- The Guardant360 CDx for the *EGFR* mutations ex19del, L858R and T790M for predicting response to osimertinib in patients with NSCLC and *KRAS* G12C for predicting response to the RAS inhibitor sotorasib, also in NSCLC.
- Therascreen KRAS RGQ PCT kit, also for predicting response to sotorasib in patients with NSCLC.

However, the FDA again added that if the specific genetic alterations associated with response to these therapeutics were not detected in blood, then a tumour biopsy should be performed to determine if the specific alterations were present.

In addition to these approved tests, several emerging therapy predictive and resistance biomarkers measurable using ctDNA have recently been reported. These include mutant *HER2* for predicting response to neratinib in metastatic breast cancer [76,77], mutant *AKT1* for predicting response to capivasertib, also in breast cancer [77], and tumour mutational burden for predicting response to immune checkpoint-based immunotherapy in advanced NSCLC [78,79,80]. Emerging resistance-conferring genetic alterations found using ctDNA include mutations in *ESR1* (codes for ER) in patients with breast cancer who develop resistance to aromatase inhibitors [81,82], reversion mutations in *BRCA1/2* genes in patients with diverse solid cancers that develop resistance to PARP inhibitors [83] and mutations in *FGFR2* in patients with cholangiocarcinoma who become resistant to FGFR inhibitor [84]. Currently, however, these resistance mechanisms are not directly actionable.

## 10. From One Gene and One Drug to Multigenes and Multidrugs

The traditional approach for analysing predictive biomarkers/companion diagnostics has involved the measurement of mutations in only one gene (usually by PCR) to select for response to a single therapeutic. However, with the decreasing costs of next generation sequencing (NGS), leading to its increased availability as well as the increasing numbers of multiple therapies for some cancer types (e.g., NSCLC and CRC), several clinical trials now require testing for mutations in multiple genes using NGS. Indeed, measurement of multiple predictive genes/companion diagnostics is currently recommended by a least one expert panel, i.e., the National Comprehensive Cancer Network (NCCN), for patients with NSCLC [45]. Until recently, most NGS analysis was performed on tissue rather than ctDNA.

One of the first reports describing the feasibility of using ctDNA as a substitute for tumour tissue was the TARGET study [85]. In part A of this study which included 100 patients with a variety of advanced cancers, Rothwell et al. [85], using a 641-gene panel, showed that successful analysis was achieved in 99% of the ctDNA samples compared with 95% for tissue. Overall, a concordance of 75% was obtained between the mutational status of the two sources. Actionable mutations were detected in ctDNA from 41 patients, of which 11 received a matching therapy as part of a phase I trial. Of these 11 patients, 4 exhibited a partial response. Unlike other reports [26,27], the turn-around times for reporting of results in this study was similar for ctDNA and tumour tissue (mean of 33 days versus 30 days, respectively).

Subsequently, large prospective phase IIa multicentre trials tested the potential clinical utility of multigene analysis of ctDNA for therapy selection in patients with advanced breast cancer [77]. In this study (plasmaMATCH), which included 1034 patients, plasma mutations in *ESR1*, *PIK3CA*, *HER2 AKT1* and *PTEN* were measured by ddPCR in all participating subjects and by the Guardant360 assay in 800 subjects. Sensitivity of the PCR ctDNA testing for mutations identified in matching tissue sequencing was 93% overall but reached 98% in samples where contemporaneous biopsies were analysed.

Patients were divided into four cohorts as follows: those with *ESR1* mutations were treated with fulvestrant; those with *HER2* mutations received neratinib, and if ER-positive also fulvestrant; those with *AKT1* mutations and ER-positive cancer received capivasertib plus fulvestrant; while those with *AKT1* mutations and were ER-negative cancer or had *PTEN* mutations were treated with capivasertib alone. After a median follow-up of 14.4 months, patients with *HER2* mutations treated with neratinib and those with ER-positive disease and *AKT* mutations treated with capivasertib plus fulvestrant reached the target number of responses, i.e., 5/20 (25%) and 4/18 (22%), respectively. According to the authors, these response rates were similar with those previously found with respective tissue assays. In contrast, findings from the other two cohorts failed to reach the pre-set target number of responses.

In another study using ctDNA multigene analysis, Nakamura et al. [86] compared the effectiveness of plasma versus tissue assays for enrolling patients with advanced gastrointestinal cancers, into clinical trials. Tissue assays were performed as part of the GI-SCREEN trial (*n* = 5743), while the ctDNA measurements were performed in patients participating in the GOZILA trial (*n* = 1787). Sequencing of ctDNA was carried using the Guardant360 assay, while tissue sequencing was performed using the OCA (v 1 and 3) method. According to the authors, the demographics and clinical characteristics of the patients were similar in the two studies, except for the prevalence of pancreatic ductal adenocarcinoma, which was higher in GOZILA trial than in GI-SCREEN (22% versus 11%).

To compare ctDNA and tissue mutation concordance, 232 patients with CRC had both assays performed. Depending on the specific gene, the overall concordance between the two sources varied between 85.0 and 100% but this increased to 97.0 to 100% for clonal mutations. Importantly, ctDNA testing was found to significantly shorten the screening duration (11 versus 33 days, *p* < 0.0001) and improve trial enrolment (9.5 versus 4.1%, *p* < 0.0001) without compromising treatment efficacy, compared to tissue testing.

The results of these trials suggest that ctDNA-based multigene mutation NGS assays have sufficient accuracy in selecting patients for participating in therapy-related clinical trials and perhaps later for widespread adoption into clinical practice.

## 11. Limitations of ctDNA Testing for Predictive Biomarkers/Companion Diagnostics

### 11.1. Limited Sensitivity for Low Volume Tumours

While the use of ctDNA versus tissue has several advantages for therapy prediction assays (Table 2), it also has several limitations (Table 3). One of the major limitations is that not all tumours, even in an advanced state, release ctDNA into the circulation. In particular, low volume tumours generally shed less ctDNA than larger samples [87]. In addition, some types of tumour such as those occurring in the brain tend to release lower levels of ctDNA into blood compared to colorectal, liver or lung lesions [87,88]. For patients with brain tumours shedding little or no detectable DNA into the bloodstream, testing for mutations in CSF might be possible [89]. Potential approaches to increase sensitivity include taking larger volumes of blood (not always possible), using ultra-low sensitive sequencing, combining mutational and methylation measurements, measurement of the corresponding mRNA (see below) or using fluids other than blood (e.g., urine for bladder cancers, CSF for brain cancers, saliva for oral cancers).

### 11.2. Interference by CHIP

Overall, the specificity of ctDNA versus tissue for actionable driver mutations is excellent, see above. However, some mutations/variants detected in plasma may not be derived from tumours but from a benign disorder known as clonal haematopoiesis of indeterminate potential (CHIP) [90]. CHIP involves clonal expansion of myeloid stem cells in the bone marrow and occurs in >10% of apparently healthy individuals > 70 years. Myeloid cells acquire mutations, especially in leukaemia-associated genes such as *DNMT3A*, *TET2*, *TP53 JAK2* and *ASXL1*. DNA containing these mutations can be shed and detected in plasma where their presence may confound the interpretation of predictive ctDNA assays [90].

The prevalence of CHIP-associated mutations/variants in patients with cancer reported in the literature has been found to vary from <1% to 62% [91,92]. This wide variation is likely to be due to method used in sequencing DNA (e.g., breadth and depth of sequencing) and/or characteristics of patients studied (e.g., previous treatments, age of patients). Fortunately, most of the CHIP-derived mutant/variant genes are not currently actionable for solid tumour, although a JAK2 inhibitor (fedratinib) has been approved by the US FDA for the treatment of the rare bone marrow disease, myelofibrosis. Strong actionable genes with predictive potential such as mutant *EGFR*, amplified or mutant *HER2* or mutant *PIK3CA* do not appear to be commonly associated with CHIP. However, mutant *KRAS,* apparently derived from CHIP, was found in ctDNA from 3/236 patients with metastatic CRC [93]. All of these patients however, received prior chemotherapy. As mentioned above, mutant *KRAS* in tissue is widely used to predict intrinsic resistance to anti-EGFR antibodies in patients with metastatic CRC. However, at present the ctDNA assay for RAS mutations is not widely used.

Evidence that CHIP may lead to misinterpretation of predictive ctDNA assays was recently reported in a study involving men with advanced prostate cancer who were undergoing plasma mutation testing for potential response to PARP inhibitors [94]. Approximately 10% of these men were found to have CHIP-related mutations in the DNA repair genes *CHEK2*, *BRCA2* and *ATM* that potentially could have led to false-positive findings. Interference of CHIP-derived genes in ctDNA assays however, can be filtered out or minimised by sequencing DNA from paired white cells. A further advantage of sequencing white cell DNA is that it can also result in germline alterations being filtered out [95].

In addition to CHIP, other lesions such as cancer precursor lesions, benign clonal expansions or incidental occult tumours unrelated to the cancer of interest could also shed mutant DNA into the circulation. There is however, little evidence that benign lesions other that CHIP release mutant DNA into the circulation.

### 11.3. Difficulties in Analysing If Mutant Actionable Gene Is Expressed 

It is sometimes forgotten that not all genes including mutated genes are consistently expressed. Thus, in a study in which 1417 different tumour types were subjected to whole exome and whole-transcriptome sequencing, 13% of the identified somatic mutation/variants genes were not found to be expressed or expressed in low levels at the mRNA level [96]. Amongst the potentially actionable genes not expressed or expressed at low levels, were *ALK* and *RET.* The *ALK* gene is translocated in 3–5% of patients with NSCLC and is a validated target for at least five approved therapeutics for this malignancy, i.e., crizotinib, ceritinib, alectinib, brigatinib, and lorlatinib [97]. *RET* gene alterations are also found in a small proportion of patients with NSCLC, but additionally, can be present in certain histological types of thyroid cancer (medullary and papillary). Selpercatinib and pralsetinib are approved therapeutics for targeting RET alteration in these malignancies [98].

Thus, to enhance the number of targetable mutations, the expression of potentially actionable genes should also be measured. Support for combined analysis was recently obtained by Sailer et al. [99] who reported that the number of patients matched for a targeted therapy increased from 39% to 55% when whole exome RNA-seq was performed in addition to DNA-seq. Although combined measurement of DNA and RNA analysis may increase, the number of actionable genes detected, we still lack suitable assays for detecting what should approximate the ideal predictive biomarker, i.e., level and/or activity of the protein drug target.

Evidence that analysing all three types of molecules can increase the number of patients matched for targeted therapies was recently obtained by Beaubier et al. [100]. These authors reported that combining measurement of DNA, mRNA alterations and immunological protein biomarkers increased the number of actionable mutations form 29.6% with DNA alone to 43.4% [100]. While DNA, mRNA and specific proteins (e.g., oestrogen receptors, HER2, PD-L1) analysis can be performed on tissue, neither mRNA or oncoproteins can be readily measured in plasma (mRNA, due to its instability and low concentration; protein because of the uncertainty of its tissue of origin when measure in blood). This limitation, however, might be minimised by using circulating tumour cells (CTC) or extracellular vesicles (EV).

### 11.4. ctDNA Not Suitable for Detecting Certain Gene Alterations 

Some actionable genetic alterations such as fusions involving large intronic sequences are best detected at mRNA level rather than using DNA [101]. For example, in 2522 cases of NSCLC that were negative for actionable genes using an FDA-cleared DNA assay (MSK-IMPACT), 232 samples had RNA-seq performed [102]. Of these, 36 cases, (15.5% of tested cases) were positive for an actionable target that was not detected at the DNA level. Importantly, 8/10 (80%) patients derived clinical benefit when their treatment was based on the molecular alteration identified at the RNA level. As mentioned above, while both DNA and RNA-seq can be performed on tissues in most cases, RNA analysis cannot be easily carried out using plasma. Again this problem might be overcome by analysing CTC or EV. Finally, ctDNA may not be suitable for detecting copy-number variant such as deletions or shallow gains, especially when its abundance is low [103].

### 11.5. ctDNA Unable to Identify Non-Genetic Mechanisms of Resistance

While most of the published literature has focused on genetic mechanisms of drug resistance, resistance can also be mediated by non-genetic alterations such as cell-type switching and stromal remodelling. Such mechanisms of resistance have been identified in CRC patients treated with the anti-EGFR antibody, cetuximab [104] and in NSCLC patients treated with EGFR TKIs [105].

## 12. Clinical Implementation of ctDNA Mutation Testing

Currently, most guidelines published by expert panels recommend ctDNA mutation testing only when tissue is unavailable or unsuitable for analysis. A possible alternative strategy is to initially perform ctDNA analysis and only reflex to tissue if the ctDNA result is negative. This is a reasonable approach given the high specificity and high positive predictive value of the plasma test. Also, according to van’t Erve et al. [106], this approach has the potential to be cost-saving compared to up-front tissue testing. Furthermore, for those patients who have a ctDNA positive result, therapy might be started earlier than waiting for a tissue result. On the contrary, waiting for a tissue result following a negative ctDNA test could delay the start of treatment. Yet a further potential strategy might be to perform combined tissue and ctDNA testing on all patients. This approach could increase the number of actionable mutations identified and thus the proportion of patients benefiting from a specific therapy [23,24,25,26,92]. However, it would be more expensive and prolong the turn-around-time for results. In addition, it is still unclear if patients who are tissue-negative but ctDNA-positive benefit from matching therapy.

## 13. Conclusions

Although tissue testing remains the gold standard for analysis of predictive biomarkers and companion diagnostics, the use of ctDNA has several attractions including ease of use and convenience, more rapid turn-around-time for reporting of results and an ability to perform serial testing. Additionally, use of ctDNA will potentially allow more patients to receive targeted therapy [23,24,25,26]. The main limitation of ctDNA testing is lower sensitivity than that available from tissue. Going forward, it will be thus important to improve sensitivity without negatively impacting on specificity. In addition, it will be important to validate (analytically and clinically) other ctDNA-based predictive biomarkers/companion diagnostics, especially *RAS* mutation testing for predicting resistance to anti-EGFR antibodies in patients with CRC. Future research should aim to increase the number of patients benefiting from matched therapies by performing mutational analysis on specific proximal fluids in addition to plasma and investigating the potential benefit of evaluating other types of liquid biopsy assays (CTC, EV) which might enable RNA analysis to be performed in addition to DNA.

## Figures and Tables

**Table 1:** Examples of predictive biomarkers/companion diagnostics for therapy decision making in patients with different advanced cancers.

Biomarker	Alteration	Therapy	Cancer(s)	LOE
ER/PR	Overexpression	Endocrine	Breast	NS
HER2	Amplification/overexpression	Anti-HER2	Breast	1
HER2	Amplification/overexpression	Anti-HER2	Gastric	1
EGFR	Mutation (ex 19 del, L858R)	EGFR TKIs	NSCLC	1
PIK3CA	Mutation	Alpelisib	Breast	1
KRAS/NRAS *	Mutation	Cetuximab, panitumumab	CRC	1
BRCA1/2	Mutation **	Olaparib, rucaparib	Prostate (castrate resistant)	1
BRCA1/2	Mutation	Rucaparib	Ovarian	1
BRCA1/2	Mutation **	Olaparib, talazoparib	Breast	2
ATM	Mutation	Olaparib	Prostate (castrate resistant)	1
ALK	Translocation	Alectinib	NSCLC	1
HER2 *	Mutation	Neratinib	Breast	3
AKT *	Mutation	Capivasertib	Breast	3
NTRK1/2/3	Fusion	Entrectinib, Larotrectinib	All solid tumours	1
FGFR2/3	Fusion	Erdafitinib	Bladder	1
FGFR2	Fusion	Cholangiocarcinoma	Pemigatinib	1
RET	Fusion	NSCLC	Pralsetinib, Selpercatinib	1
RET	Fusion	Thyroid	Pralsetinib, Selpercatinib	1
MSI-H *, TMB *		Immune checkpoint inhibitors	Multiple	1

LOE, level of evidence as specified in OncoKB list of actionable genes. ER/PR, oestrogen and progesterone receptors; NS, not stated (but should be LOE 1): NSCLC, non-small-cell lung cancer; CRC, colorectal cancer; CR, castrate resistant; MSI-H, microsatellite instability high; ICI, immune checkpoint inhibitors. * Tests do not have an US FDA approved assay, ** germline mutation. Data summarised from references [13,14,15,16] and https://www.oncokb.org/actionableGenes (accessed on 14 June 2021). List of predictive biomarkers/companion diagnostics is not meant to be comprehensive.

**Table 2:** Limitations of tissue and corresponding advantages of ctDNA for detecting predictive biomarkers/companion diagnostics.

Limitations of Tissue	Advantages of ctDNA	Reference(s)
Inconvenient for patients	More convenient and acceptable	
Invasive and risky	Minimally invasive and little risk	
Access may not be possible in some patients	Almost always accessible	
May not fully capture tumour heterogeneity	More likely to capture tumour heterogeneity especially with acquired resistance	[22]
Can miss some actionable mutations due to tumour heterogeneity	More actionable mutations detected	[23,24,25,26]
Relatively long turn-around-time	Shorter turn-around-time	[26,27]
Difficult to repeat or perform serial assays	Minimal problem in performing serial assays	
Relatively expensive	Less expensive	[28,29]

**Table 3:** Disadvantages of ctDNA assays for detecting predictive biomarkers/companion compared with tissue assays.

Less Sensitive, May Miss Tissue-Positive Samples
Susceptible to misinterpretation due to CHIP *
Unable to determine if detected mutant gene is expressed
Unable to detect certain potentially actionable gene fusions
Unable to identify non-genetic mechanisms of resistance
Only a small number have validated or approved assays
Requires deeper DNA sequencing

* CHIP, clonal haematopoiesis of indeterminate origin, can be eliminated or minimised by simultaneously sequencing matching white cell DNA or performing mutation analysis on plasma low molecular weight DNA fragments.

## Data Availability

Not applicable.
